# Meeting report from the second EurocanPlatform summer school on translational cancer research, Portugal, 20–24 October 2014

**DOI:** 10.3332/ecancer.2015.512

**Published:** 2015-02-19

**Authors:** Grazyna Lipowska-Bhalla

**Affiliations:** Targeted Therapy Group, Institute of Cancer Sciences, Manchester Cancer Research Centre, University of Manchester, Manchester Academic Health Sciences Centre, Manchester M20 4BX 2, UK

**Keywords:** EurocanPlatform summer school 2014, meeting report, translational cancer research

## Abstract

The second EurocanPlatform summer school was held in Algarve, Portugal and attracted scientists, clinicians and pathologists with a common interest in cancer research to discuss the latest developments and challenges in the field. The meeting focused on translational cancer research and also included lectures, workshops and discussions, which covered all aspects of the translational research continuum, from early detection through treatment to survivorship. The rate of new cancer cases and cancer mortality increases every year. Although the last decade witnessed enormous progress in understanding cancer biology and the development of new therapies, the efficacy of these therapies is challenged by cancer resistance. It clearly suggests that new druggable targets are required and their translation from laboratory to bedside must be faster and more efficient to improve survival rates and standards of care.

The second EurocanPlatform summer school on translational cancer research was held in Portugal, and in this meeting, leading oncology scientists, basic researchers, clinicians, pathologists and patient organization representatives from Europe and the USA participated to discuss about latest developments and challenges in the field of translational cancer research.

The five-day programme included lectures, workshops and discussions, which covered all aspects of the translational research continuum, from early detection through treatment to survivorship. The meeting created a unique interactive platform for young basic scientists and clinicians to connect with leading figures in cancer research and provided a forum for stimulating discussions. About 20 EurocanPlatform-OECI travel fellowships were granted to talented PhD students, early-stage postdoctoral fellows and clinicians to attend the meeting.

The meeting was organized by members of the EurocanPlatform consortium in collaboration with *e*cancer, the German Cancer Consortium (DKFZ), the European Network for Cancer Research in Children and Adolescents (ENCCA), the European CanCer Organization (ECCO) and the Organization of European Cancer Institutes (OECI). The EurocanPlatform consortium is a European Commission-funded Network of Excellence that brings together 28 European cancer research centres and organizations to advance cancer research and treatment (www.eurocanplatform.eu).

The meeting hosted several scientific sessions spanning the innovative aspects of cancer biology, research areas driving preventive and clinical research, bridging preclinical and clinical research, clinical and late translational cancer research and personalized medicine. The meeting also hosted talks given by Francesco de Lorenzo, President of the European Cancer Patient Coalition (ECPC), who presented the patients’ perspective on the development of personalized cancer treatments, and by Bengt Jönsson from the Stockholm School of Economics who discussed the role of health economics in cancer research.

The meeting began with invited speaker Isabelle Soerjomataram (International Agency for Research on Cancer, Lyon, France) who gave an overview of the current global burden of cancer and a future prediction of 22 million new cancer cases and over 13 million deaths from cancer occurring annually by 2030, based on current trends of cancer incidence and mortality. Richard Schilsky (American Society of Clinical Oncology, USA) discussed cancer evaluation and treatment in the genomic era reviewing the traditional clinical approaches and highlighting the importance of genomic information in formulating an appropriate assessment and treatment strategy for patients with cancer. Ioanna Keklikoglou (Swiss Federal Institute of Technology, Lausanne, Switzerland) presented the concept of ‘hallmarks of cancer’ [1, 2] and indicated combinatorial targeting of multiple hallmarks of cancer as the main strategy for developing more effective and durable cancer therapies. Interactions between the tumour and its microenvironment and the clinical implications of these interactions were addressed by Mari Grazia Daidone (National Institute of Cancer, Milan, Italy). Thomas Tursz (Institute Gustave Roussy, Villejuif, France) gave insight into personalized cancer treatments and reviewed successes and failures of cancer-targeted therapies over recent decades.

The genetic and epigenetic aspects of cancer development and progression were also highlighted during the meeting. Christopher Lord (Institute of Cancer Research, London, UK) gave an insight into DNA damage and repair mechanisms and discussed targeting these mechanisms in cancer. Alvis Brazma (European Molecular Biology Laboratory, European Bioinformatics Institute, Cambridge, UK) discussed about integrative analysis of cancer genome and transcriptome, which could be helpful to explore the underlying genomic architecture of cancer and uncover unknown mutations and abnormal pathways that represent potential targets for developing new cancer therapies. Zdenko Herceg (International Agency for Research on Cancer, Lyon, France) addressed epigenome deregulation in cancer and the epigenetics-based strategies for cancer treatment and prevention.

The post-translational modifications (PTM) frequently altered in tumour cells were also discussed by several speakers. Pauline Rudd (National Institute for BioProcessing Research and Training, Dublin, Ireland) discussed the role of altered glycosylation pathways in promoting tumour metastasis and stressed the role of profiling glycosylation changes in cancer patients as useful clinical markers for patient stratification and determining pathways involved in the development and progression of the disease. Yifat Merbl (Weizmann Institute of Science, Rehovot, Israel) gave insight into ubiquitin and ubiquitin-like protein modifications and presented results from work on developing new approaches for profiling PTM on a proteome-wide scale.

Cancer therapy resistance, one of the main problems hampering cancer-targeted therapy, was highlighted by Sven Rottenberg (Netherlands Cancer Institute, Amsterdam, the Netherlands) who presented how molecular mechanisms underlying drug resistance can be studied in mouse models. Genetically modified mice with tissue-specific deletion of BRCA1 or BRCA2 in mammary epithelium spontaneously develop breast cancer which closely mimics the disease in humans [3]. Loss of BRCA1 or BRCA2 function results in defective homologous recombination (HR) and failure to repair double-strand DNA breaks, whereas repair of single-strand brakes is mediated by poly (ADP-ribose) polymerase 1 (PARP1). BRCA-deficient cancers are sensitive to PARP1 inhibitor olaparib [4, 5]. PARP1 inhibition leads to unrepaired single-strand DNA breaks that eventually turn into double-strand breaks that also cannot be repaired due to failure of BRCA1 or BRCA2 and lead to cell death. However, long-term olaparib treatment results in developing resistance that can be driven by number of different mechanisms. Study on mouse model for BRCA-deficient breast cancer revealed that olaparib resistance was driven by up-regulation of Abcb1a/b genes encoding P-glycoprotein and could be reversed by inhibition of P-glycoprotein [4]. However, subsequent pre-clinical study reported that indeed inactivation of P-glycoprotein increased the long-term response of BRCA-deficient breast cancer to olaparib, but these tumours eventually acquired resistance to the therapy. This resistance involved a new mechanism in which homologous recombination was partially restored by loss of p53-binding protein 1 (53BP1) [6]. These studies show that pre-clinical evaluation of anticancer drugs in animal models can deliver clinically relevant information and facilitate development of more efficient therapeutic strategies.

According to cancer research carried out in United Kingdom, more than one of three people will develop cancer, but more than four of 10 cancer cases could be prevented. Pierre Hainaut (University of Grenoble, France) discussed translation of cancer biology into preventive strategies with the main recommendations include reducing the exposure to cancer-causing agents, healthy diet, keeping active, maintaining healthy body weight and avoiding certain infections such as hepatitis B virus or human papilloma virus.

In order to make preventive programmes effective and improve cancer treatment and monitoring, appropriate biomarkers are required. In the bridging pre-clinical and preventive/clinical research session, Anne-Lise Börresen-Dale (Oslo University Hospital, Oslo, Norway) reported on the development of biomarkers for early detection and risk of relapse in breast cancer. Malignant transformation is a slow process and usually takes long time before single abnormal cell gives rise to palpable tumour. This creates a window of opportunity for early detection. However, appropriate markers for transformation need to be identified. The first step to know initial phase of breast carcinogenesis is to understand variability in healthy breast. Two clusters with different characteristics were identified from a study describing 9,767 genes expression profile in breast biopsies taken from 79 healthy women [7]. Samples in cluster 1 had more undifferentiated characteristic compared to cluster 2 with stromal and stem-like gene signature. Interestingly, these samples shared many features with claudin-low breast cancer. Among 2,621 genes differentially expressed between cluster 1 and 2, about 1,516 genes were up-regulated in cluster 1, and these were associated with vascular development, glucose metabolism, cell motion and regulation of inflammatory response, all being relevant to carcinogenesis. The down-regulated genes were enriched for genes associated with actin-binding, adherence, cytoskeleton and plasma membrane. Although this study is limited by the low number of women included, it suggests that the woman falling in cluster 1 may possibly be at higher risk of developing claudin-low and basal-like breast cancer. Increased breast cancer incidence in the family history of women classified in this cluster seems to support this hypothesis. Another study focused on identification of gene expression signature associated with malignant transformation of ductal carcinoma *in situ* (DCIS). DCIS is a non-malignant lesion, and it is estimated that one in three women in their age of 40 have DCIS or closely related lesion that potentially can rapidly transform into malignant form of DCIS. In this study, gene expression signature of 31 ductal carcinomas *in situ* was investigated and compared against 36 invasive breast carcinomas, 42 mixed lesions (mixture of invasive carcinoma with DCIS component) and six healthy breast samples [8]. Clustering of DCIS samples using 826 most variable genes identified two distinct DCIS subgroups with one of these groups exhibiting phenotype more similar to invasive breast cancer than to non-malignant samples. This phenotype was independent of oestrogen receptor and HER2 status as well as histological grade of DCIS. Similar to invasive breast carcinoma, genes up-regulated in invasive-like DCIS subgroup were enriched for extracellular matrix (ECM)-related proteins and proteins associated with epithelial–mesenchymal transition (EMT), both playing an important role in malignant transformation process. Distinguishing between non-invasive and invasive-like DCIS could facilitate assessment of the risk and early diagnosis of malignant breast cancer.

Several speakers addressed the role of genomics and proteomics in cancer research with René Bernards (Netherlands Cancer Institute, Amsterdam, the Netherlands) discussing the role of omics technologies for the development of predictive cancer medicine and Janne Lehtiö (Karolinska Institutet, Stockholm, Sweden) giving an overview of recent developments in cancer proteomics and indicating a crucial role of proteomics in understanding cancer resistance to targeted therapies and identification of novel predictive markers.

Radiotherapy, the standard treatment used in nearly 50% of all patients with cancer, was discussed by Michael Bauman (University of Technology, Dresden, Germany) who gave a mechanistic insight into radiotherapy-induced tumour control and highlighted the recent progress in radiotherapy technology and its impact on clinical efficacy. Cancer immunotherapy, the Science breakthrough of the year 2013 [9], was presented by Rolf Kiessling (Karolinska Institutet, Stockholm, Sweden) who described the current status of immunotherapy in malignant melanoma and outlined the future challenges in the field, while Jonas Bergh (Karolinska Institutet, Stockholm, Sweden) discussed side effects of conventional and targeted cancer therapies.

Current challenges in paediatric oncology were highlighted by several speakers with Giuseppe Barone (The Institute of Cancer Research and The Royal Marsden Hospital, London, UK) discussing the difficulties in paediatric drug development with emphasis on early phase clinical trials and Olaf Witt (German Cancer Research Centre, Heidelberg, Germany) highlighting the challenges of developing targeted therapies in paediatric brain tumours. Cornelia Eckert (Charité University of Berlin, Germany) presented how some of the challenges in paediatric oncology can be overcome by the identification of new predictive markers and molecular targets using latest technologies and gave examples of their successful integration into therapy optimization concepts.

The need for multilevel partnership and infrastructure in advancing translational cancer research was also widely discussed during the meeting. Denis Lacombe (European Organization for Research and Treatment of Cancer, Brussels, Belgium) discussed the current challenges in clinical research and stressed the requirement for new approaches and partnership models for biomarker-based clinical research, while Peter Riegman (Erasmus Medical Centre, Rotterdam, Netherlands) indicated the fundamental role of dedicated biobanking infrastructure in translational cancer research. Gunnar Säter (Oslo University Hospital, Oslo, Norway) gave insight into the complexity of the academia—pharmaceutical industry relationship and highlighted the increasing role of academia in the drug development process. Norbert Graf (Saarland University, Homburg, Germany) gave an overview of the p-medicine project that, similarly to the EurocanPlatform programme, is cooperation between 19 European and Japanese institutions whose common effort is to pave the way for more individualized therapies for patients with cancer.

Several speakers addressed the increasing role of imaging technologies in every facet of cancer care. Peter Dean (International Agency for Research on Cancer, Lyon, France) discussed the impact of diagnostic imaging findings on the success of interdisciplinary breast cancer management. Hedvig Hricak (Memorial Sloan-Kettering Cancer Centre, New York, USA) described how advances in imaging technologies transformed cancer treatment and underlined the critical role of molecular imaging (MI) in integrated diagnostic in which rapid *in vivo* assessment of selected biomarkers with MI will complement laboratory diagnostic.

A final day workshop focused specifically on personalized medicine with Carlos Caldas (University of Cambridge, UK) discussing molecular stratification of breast cancer, Mathew Garnett (Wellcome Trust Sanger Institute, Cambridge, UK) describing how cancer cell models can be used as a therapeutic biomarker discovery platform, James Brenton (University of Cambridge, UK) presenting tumour heterogeneity as a prognostic/predictive marker, and Peter Collins (University of Cambridge, UK) discussing the merge of classical and molecular histopathology. Alberto Bardelli (University of Turin, Italy) demonstrated the application of liquid biopsies for monitoring treatment response in colorectal cancer, while Wilbert Zwart (Netherlands Cancer Institute, Amsterdam, the Netherlands) presented the profiling of ERα chromatin-binding sites for the identification of new biomarkers for tailoring treatment in patients with breast and endometrial tumours.

## Conclusion

In summary, the meeting highlighted the fast pace of progress in translational cancer research and stressed the need for new strategies and collaborations to surmount the increasing challenges in bringing cancer therapies from the bench to bedside. To achieve this, discussants agreed that key areas, such as prevention, early detection, treatment and research, need to be balanced and coordinated. New preventive, research and clinical strategies are required along with new forms of partnership and collaboration between academia, clinical centres and the pharmaceutical industry as well as regulatory bodies and patient organizations to facilitate faster delivery of new cancer therapies and standards of care.
